# The gene spectrum of thalassemia in Yangjiang of western Guangdong Province

**DOI:** 10.3389/fgene.2023.1126099

**Published:** 2023-02-13

**Authors:** Hong-Feng Liang, Wei-Min Liang, Wen-Guang Xie, Fen Lin, Li-Li Liu, Lie-Jun Li, Yi-Yuan Ge, Min Lu, Yu-Wei Liao, Guang-Kuan Zeng, Jin-Xiu Yao, Jing-Wei Situ, Li-Ye Yang

**Affiliations:** ^1^ Precision Medical Lab Center, People’s Hospital of Yangjiang, Yangjiang, Guangdong, China; ^2^ Medical Laboratory, Women and Children Hospital of Yangjiang, Yangjiang, Guangdong, China; ^3^ Precision Medical Lab Center, Chaozhou Central Hospital Affiliated to Southern Medical University, Chaozhou, Guangdong, China; ^4^ Guangdong Hybribio Limited Corporation, Chaozhou, Guangdong, China; ^5^ Laboratory Medical Center, People’s Hospital of Yangjiang, Yangjiang, Guangdong, China

**Keywords:** thalassemia, gene testing, genotype, China, Guangdong Province

## Abstract

**Background:** Thalassemia presents a higher incidence in southern China. The objective of this study is to analyze the genotype distribution of thalassemia in Yangjiang, a western city of Guangdong Province in China.

**Methods:** The genotypes of suspected cases with thalassemia were tested by PCR and reverse dot blot (RDB). Unidentified rare thalassemia genotypes of the samples were further ascertained by PCR and direct DNA sequencing.

**Results:** Among 22467 suspected cases with thalassemia, 7658 cases were found with thalassemia genotypes using our PCR-RDB kit. Among these 7658 cases, 5313 cases were found with α-thalassemia (α-thal) alone, --^SEA^/αα was the most common genotype, accounting for 61.75% of α-thal genotypes, and the following mutations were found: α^3.7^/αα, -α^4.2^/αα, α^CS^α/αα, α^WS^α/αα, and α^QS^α/αα. A total of 2032 cases were found with β-thalassemia (β-thal) alone. β^CD41-42^/β^N^, β^IVS−II−654^/β^N^, and β^−28^/β^N^ accounted for 80.9% of all β-thal genotypes, and the following genotypes were found: β^CD17^/β^N^, β^CD71-72^/β^N^, and β^E^/β^N^. Compound heterozygotes of β-thal and β-thalassemia homozygotes were identified in 11 and five cases, respectively, in this study. α-thal combined with β-thal was identified in 313 cases, showing 57 genotype combinations of the coincidence of both Hb disorders; one extreme patient had a genotype of --^SEA^/α^WS^α and β^CD41-42^/β^−28^. In addition, four rare *α*-mutations (--^THAI^, HKαα, Hb Q-Thailand, and CD31 AGG>AAG) and six rare β-mutations (CD39 CAG>TAG, IVS-Ⅱ-2 (−T), −90(C>T), Chinese ^G^γ^+^(Aγδβ)0, CD104 (-G), and CD19 A>G) were also found in this study population.

**Conclusion:** This study provided detailed genotypes of thalassemia in Yangjiang of western Guangdong Province in China and reflected the complexity of genotypes in this high-prevalence region, and this would be valuable for diagnosis and counseling for thalassemia in this area.

## 1 Introduction

Thalassemia is one of the most common human genetic disorders that are characterized by decreased or absent synthesis of one or more hemoglobin polypeptide chains. Thalassemia carriers are found at variable frequencies in all subtropical and tropical populations. Southern China has a high incidence of thalassemia, with a carrier frequency of 3%–24% (Weatherall, 1997). Of all hemoglobin disorders, *α*-thalassemia (α-thal) and β-thalassemia (β-thal) are the most widely distributed, and therefore many individuals in these areas have complex combinations of these variants (Weatherall, 1997).

Clinical manifestations of α-thal range from asymptomatic cases to patients with lethal hydrops fetalis syndrome. Patients with β-thal homozygote present severe thalassemia, while the patients with compound heterozygosity, such as β-thal/Hb E, present variable severity of anemia (Weatherall, 2004). Severe thalassemia poses a major public health concern in areas where thalassemia is endemic.

Yangjiang, with an area of approximately 7,813.4 km^2^ and a population of 2.7 million, includes two counties and two prefectures, locates to the west of Guangdong Province in southern China, and borders Maoming to the southwest, Yunfu to the north, and Jiangmen to the east. All these places are known to be the regions of prevalence of thalassemia (Xu et al., 2004; Yin et al., 2014).

A previous study showed that the frequency of thalassemia in a county in Yangjiang was 20%, and about 5% of the population was β-thal carriers, which was the highest in Guangdong Province (Yin et al., 2014). Thalassemia has different carrier frequencies in different regions, with strong genetic heterogeneity and ethnic differences. To date, the gene distribution and complexity of thalassemia in whole Yangjiang have not been reported. Therefore, it is necessary to clarify the true distribution of various thalassemia genotypes and their co-inheritance patterns in this area of southern China.

In this study, we performed genotyping of the six α-thal defects [--^SEA^, -α^3.7^, -α^4.2^, α^CS^ (Constant Spring), α^QS^ (Quong Sze), and α^WS^ (Westmead)] and 19 common β-thal mutations in two hospital-based populations in Yangjiang City. An unidentified genotype of the samples was further ascertained by PCR and DNA sequencing. We aimed to get a better understanding of the gene distribution of thalassemia and its co-inheritance patterns in this area, which was a prerequisite to perform appropriate prevention and control programs.

## 2 Materials and methods

### 2.1 Study population

This study was performed in two local hospitals (People’s Hospital of Yangjiang and Women and Children Hospital of Yangjiang) between September 2014 and July 2021 in Yangjiang. The majority of the study subjects were couples who received thalassemia screening and those subjects either with suspicion of a thalassemia trait or with clinically recognized thalassemia syndrome of different degrees of severity. About one-third of the study subjects received thalassemia gene diagnosis directly for convenience; they did not receive blood routine test and Hb electrophoresis. Amniotic fluid, umbilical cord blood, and fetal villi were collected from 21 fetuses for prenatal diagnosis. All studies were approved by the Ethics Committee of People’s Hospital of Yangjiang and Women and Children Hospital of Yangjiang. All the patients or their guardians signed the informed consent forms.

### 2.2 Hematological analysis

A measure of 2–3 mL of peripheral blood samples of EDTA anticoagulants was taken from 22446 subjects for screening and genetic diagnosis of thalassemia. Hematological data of these subjects were obtained using an automated blood cell counter and by Hb electrophoresis. Subjects with reduced mean corpuscular volume (MCV<82 fl), reduced mean corpuscular hemoglobin (MCH<27 pg), and HbA_2_<2.5% were considered possible α-thal carriers. Subjects with MCV<82 fl, MCH<27 pg, and HbA_2_>3.5% were considered possible β-thal carriers. Some subjects who had undergone thalassemia screening in other hospitals did not receive the blood routine test and were only demanded for molecular diagnosis. Amniotic fluid, umbilical cord blood, and fetal villi were obtained from 21 fetuses for thalassemia gene diagnosis. Totally, 22467 samples were characterized by molecular diagnosis.

### 2.3 α/β-Thalassemia mutation analysis

Genomic DNA was extracted from all peripheral blood leukocytes, umbilical cord blood, fetal villi, and amniotic fluid. PCR and reverse dot blot (RDB) were used to detect the three commonly known α-thal deletions (--^SEA^, -α^3.7^, and -α^4.2^) and three known point mutations (Hb CS (α^CS^α) *HBA2*: c.427T>C; Hb QS (α^QS^α), *HBA2*: c.377T>C; Hb WS (α^WS^α), and *HBA2*: c.369C>G) responsible for α-thal, and 19 known β-thal mutations most commonly seen in the Chinese population [IVS-II-654 (C>T), *HBB*: c.316–197C>T; codons 41/42 (‒TCTT), *HBB*: c.126_129delCTTT; codon 17 (A>T), *HBB*: c.52A>T; −28 (A>G), *HBB*: c.‒78A>G; codon 26 (G>A), *HBB*: c.79G>A; codons 71/72 (+A), *HBB*: c.216_217insA; codons 27/28 (+C), *HBB*: c.84_85insC; −29 (A>G), *HBB*: c.‒79A>G; IVS-I-1 (G>A, G>T), *HBB*: c.92 + 1G>A, c.92 + 1G>T; codons 14/15 (+G), *HBB*: c.45_46insG; codon 43 (G>T), *HBB*: c.130G>T; Cap (-AAAC, A>C), *HBB*: c.-11_-8delAAAC; IVS-I-5 (G>C), *HBB*: c. 92 + 5G>C; codon 31 (‒C), *HBB*: c.94delC; initiation codon ATG>AGG, *HBB*: c.2T>G; −32 (C>A), *HBB*: c.‒82C>A; −30 (T>C), and *HBB*: c.‒80T>C] were also identified (Liang et al., 2022). All genotyping was analyzed using a thalassemia gene chip kit (Guangdong Hybribio Limited Corporation, Chaozhou, China), as described in our previous study (Liang et al., 2022).

Those cases with abnormal capillary electrophoresis behavior or thalassemia traits which could not be identified by this gene chip kit were further tested by gap-PCR and PCR sequencing based on their primary gene analysis result (Liang et al., 2022), and anti-3.7 and anti-4.2 α-globin gene triplications were identified by single-tube multiplex PCR (Wang et al., 2005). Only a small number of suspected cases with rare variants were further tested.

### 2.4 Statistical analysis

Statistical analysis was performed on the genotypic frequency of thalassemia by using the percentage method.

## 3 Results

First, genetic diagnosis of all 22467 possible thalassemia samples identified 19 known β-thal mutations and six kinds of α-thal mutations using a kit (Liang et al., 2022). We detected six common mutations that cause α-thal and 12 common mutations responsible for β-thal; five β-thal mutations of 19 known β-thal mutations were not detected by our kit (Tables 1, 2). Among 22467 possible thalassemia cases, 7658 cases were identified with thalassemia genotypes, including 5313 cases were identified with α-thal alone (Table 1), 2032 cases were identified with β-thal alone (Table 2), and 313 cases were identified with α-thal combined with β-thal (Table 3).

**TABLE 1:** Spectrum of α-thalassemia mutations alone.

Genotype	Case (n)	Phenotype	Frequency (%)
α-thalassemia heterozygote			
--^SEA^/αα	3281	α^0^/α	61.75
-α^3.7^/αα	831	α^+^/α	15.64
-α^4.2^/αα	341	α^+^/α	6.42
α^WS^α/αα	161	α^+^/α	3.03
α^QS^α/αα	126	α^+^/α	2.37
α^CS^α/αα	178	α^+^/α	3.35
Compound heterozygote			
--^SEA^/-α^3.7^	216	Hb H	4.07
--^SEA^/-α^4.2^	64	Hb H	1.20
--^SEA^/α^CS^α	42	Hb H	0.79
--^SEA^/α^WS^α	14	Hb H	0.26
--^SEA^/α^QS^α	5	Hb H	0.09
-α^3.7^/α^WS^α	7	α^+^/α^+^	0.13
-α^3.7^/-α^4.2^	12	α^+^/α^+^	0.23
-α^4.2^/α^QS^α	4	α^+^/α^+^	0.08
-α^3.7^/α^CS^α	7	α^+^/α^+^	0.13
-α^4.2^/α^WS^α	2	α^+^/α^+^	0.04
α^QS^α/α^CS^α	1	α^+^/α^+^	0.02
-α^3.7^/α^QS^α	3	α^+^/α^+^	0.06
Homozygote			
-α^3.7^/-α^3.7^	13	α^+^/α^+^	0.24
--^SEA^/--^SEA^	3	α^0^/α^0^	0.06
α^QS^α/α^QS^α	1	α^+^/α^+^	0.02
-α^4.2^/-α^4.2^	1	α^+^/α^+^	0.02
Total	5313		100

**TABLE 2:** Spectrum of β-thalassemia mutations alone.

Genotype	Case (n)	Phenotype	Frequency (%)
β-thalassemia heterozygote			
β^CD41-42^/β^N^	881	β^0^/β^N^	43.36
β^IVS−II−654^/β^N^	471	β^+^/β^N^	23.18
β^−28^/β^N^	292	β^+^/β^N^	14.37
β^CD17^/β^N^	172	β^0^/β^N^	8.46
β^CD71-72^/β^N^	85	β^0^/β^N^	4.18
β^−29^/β^N^	22	β^+^/β^N^	1.08
β^CD27-28^/β^N^	27	β^0^β^N^	1.33
β^E^/β^N^	32	β^+^β^N^	1.57
β^IVS−I−1^/β^N^	21	β^0^β^N^	1.03
β^Cap^/β^N^	2	β^+^β^N^	0.10
β^CD14-15^/β^N^	3	β^0^β^N^	0.15
β^CD43^/β^N^	8	β^0^β^N^	0.39
Compound heterozygote			
β^IVS−II−654^/β^−28^	4	β^+^/β^+^	0.20
β^CD27-28^/β^−28^	1	β^0^/β^+^	0.05
β^CD41-42^/β^−28^	2	β^0^/β^+^	0.10
β^IVS−II−654^/β^CD71-72^	1	β^+^/β^0^	0.05
β^CD17^/β^−28^	1	β^0^/β^+^	0.05
β^CD41-42^/β^CD17^	1	β^0^/β^0^	0.05
β^CD41-42^/β^E^	1	β^0^/β^+^	0.05
Homozygote			
β^CD17^/β^CD17^	2	β^0^/β^0^	0.10
β^IVS−II−654^/β^IVS−II−654^	1	β^+^/β^+^	0.05
β^CD41-42^/β^CD41-42^	2	β^0^/β^0^	0.10
Total	2032		100

**TABLE 3:** Genotype distribution of β-thal combined with α-thal mutations.

α-chain mutation	β-chain mutation	Phenotype	Case (n)
--^SEA^ */*αα	β^−28^ */*β	α^0^α/β^+^β^N^	38
--^SEA^/αα	β^CD17^/β	α^0^α/β^0^β^N^	10
--^SEA^/αα	β^CD41-42^/β	α^0^α/β^0^β^N^	83
--^SEA^/αα	β^CD71-72^/β	α^0^α/β^0^β^N^	6
--^SEA^/αα	β^IVS−II−654^/β	α^0^α/β^+^β^N^	22
--^SEA^/-α^3.7^	β^−28^/β	Hb H/β^+^β^N^	2
--^SEA^/-α^3.7^	β^CD41-42^/β	Hb H/β^0^β^N^	3
--^SEA^/-α^3.7^	β^CD17^/β	Hb H/β^0^β^N^	1
--^SEA^/-α^3.7^	β^IVS−II−654^/β	Hb H/β^+^β^N^	8
--^SEA^/αα	β^CD27-28^/β	α^0^α/β^0^β^N^	2
--^SEA^/αα	β^−29^/β	α^0^α/β^+^β^N^	2
--^SEA^/αα	β^CD43^/β	α^0^α/β^0^β^N^	3
--^SEA^/αα	β^IVS−I−1^/β	α^0^α/β^0^β^N^	2
--^SEA^/αα	β^E^/β	α^0^α/β^+^β^N^	3
--^SEA^/α^WS^α	β^CD17^/β	Hb H/β^0^β^N^	2
-α^3.7^/-α^4.2^	β^CD41-42^/β	α^+^α^+^/β^0^β^N^	2
-α^3.7^/αα	β^CD14-15^/β	α^+^α/β^0^β^N^	1
-α^3.7^/αα	β^CD17^/β	α^+^α/β^0^β^N^	11
-α^3.7^/αα	β^−28^/β	α^+^α/β^+^β^N^	6
-α^3.7^/αα	β^CD41-42^/β	α^+^α/β^0^β^N^	22
-α^3.7^/αα	β^IVS−II−654^/β	α^+^α/β^+^β^N^	10
-α^3.7^/αα	β^CD71-72^/β	α^+^α/β^0^β^N^	2
-α^3.7^/αα	β^E^/β	α^+^α/β^+^β^N^	1
-α^4.2^/αα	β^CD27-28^/β	α^+^α/β^0^β^N^	1
-α^4.2^/αα	β^CD71-72^/β	α^+^α/β^0^β^N^	1
-α^4.2^/αα	β^E^/β	α^+^α/β^+^β^N^	1
-α^4.2^/αα	β^−28^/β	α^+^α/β^+^β^N^	5
-α^4.2^/αα	β^CD41-42^/β	α^+^α/β^0^β^N^	7
-α^4.2^/αα	β^IVS−II−654^/β	α^+^α/β^+^β^N^	2
-α^4.2^/αα	β^IVS−I−1^/β	α^+^α/β^0^β^N^	1
-α^4.2^/αα	β^CD43^/β	α^+^α/β^0^β^N^	1
α^CS^α/α^CS^α	β^CD41-42^/β	α^+^α^+^/β^0^β^N^	1
α^CS^α/αα	β^−28^/β	α^+^α/β^+^β^N^	3
α^CS^α/αα	β^CD17^/β	α^+^α/β^0^β^N^	1
α^CS^α/αα	β^CD41-42^/β	α^+^α/β^0^β^N^	2
α^CS^α/αα	β^IVS−II−654^/β	α^+^α/β^+^β^N^	1
α^QS^α/αα	β^CD17^/β	α^+^α/β^0^β^N^	1
α^QS^α/αα	β^−28^/β	α^+^α/β^+^β^N^	1
α^QS^α/αα	β^CD41-42^/β	α^+^α/β^0^β^N^	4
α^WS^α/αα	β^CD17^/β	α^+^α/β^0^β^N^	2
α^WS^α/αα	β^−28^/β	α^+^α/β^+^β^N^	3
α^WS^α/αα	β^CD41-42^/β	α^+^α/β^0^β^N^	11
α^WS^α/αα	β^IVS−II−654^/β	α^+^α/β^+^β^N^	3
α^CS^α/αα	β^−28^/β	α^+^α/β^+^β^N^	1
α^QS^α/αα	β^−28^/β	α^+^α/β^+^β^N^	1
α^QS^α/αα	β^CD41-42^/β	α^+^α/β^0^β^N^	1
α^WS^α/αα	β^CD41-42^/β	α^+^α/β^0^β^N^	3
α^WS^α/αα	β^IVS−I−1^/β	α^+^α/β^0^β^N^	1
α^WS^α/αα	β^IVS−II−654^/β	α^+^α/β^+^β^N^	1
-α^3.7^/α^WS^α	β^CD41-42^/β	α^+^α^+^/β^0^β^N^	3
--^SEA^/α^CS^α	β^E^/β	Hb H/β^+^β^N^	2
--^SEA^/-α^4.2^	β^CD41-42^/β	Hb H/β^0^β^N^	2
--^SEA^/αα	β^−28^/β^CD17^	α^0^α/β^+^β^0^	1
--^SEA^/α^QS^α	β^IVS−II−654^/β	Hb H/β^+^β^N^	1
--^SEA^/α^WS^α	β^CD41-42^/β^−28^	Hb H/β^0^β^+^	1
--^SEA^/-α^4.2^	β^−28^/β	Hb H/β^+^β^N^	1
Total			313

Amniotic fluid (19 cases), umbilical cord blood (one case), and fetal villi (one case) were obtained from 21 fetuses for thalassemia gene diagnosis. They were identified as --^SEA^/--^SEA^ (three cases), --^SEA^/αα (five cases), --^SEA^/-α^4.2^ (one case), -α^4.2^/αα (one case), αα/αα (two cases), β^CD41-42^/β^CD41-42^ (one case), β^CD41-42^/β^IVS−II−654^ (one case), β^IVS−II−654^/β (two cases), β^CD41-42^/β (two cases), β^CD17^/β (one case), and β/β (two cases).

Among the 5313 α-thal cases, --^SEA^/αα was the most common genotype, accounting for 61.75% (3281/5313) of α-thal genotypes, and the following genotypes were identified: -α^3.7^/αα (15.64%, 831/5313), -α^4.2^/αα (6.42%, 341/5313), α^CS^α/αα (3.35%, 178/5313), α^WS^α/αα (3.03%, 161/5313), and α^QS^α/αα (2.37%, 126/5313). Compound heterozygotes of α-thal were found in 377 cases, and Hb H disease was found in 341 cases. As expected, the most common genotypes of patients with Hb H disease were --^SEA^/-α^3.7^ (63.34%, 216/341) and --^SEA^/-α^4.2^ (18.77%, 64/341). --^SEA^/α^CS^α, --^SEA^/α^wS^α, and --^SEA^/α^QS^α were also encountered in 42, 14, and 5 cases, respectively, in our study. In total, 18 cases were homozygotes, including 13 cases of -α^3.7^/-α^3.7^, one case of -α^4.2^/-α^4.2^, one case of α^QS^α/α^QS^α, and three cases of Hb Bart’s hydrops fetalis syndrome with the genotype of --^SEA^/--^SEA^ (Table 1).

Of the study subjects, 2032 cases were β-globin mutation alone (Table 2). In total, 22 different genotypes were detected, including 2016 heterozygotes, 11 compound heterozygotes, and five homozygotes, accounting for 99.21%, 0.54%, and 0.25%, respectively (Table 2). β^CD41-42^/β^N^ (43.36%, 881/2032), β^IVS−II−654^/β^N^ (23.18%, 471/2032), and β^−28^/β^N^ (14.37%, 292/2032) accounted for 80.9% (1644/2032) of all β-thal genotypes, and the following mutations were found: β^CD17^/β^N^, β^CD71-72^/β^N^, and β^−29^/β^N^. The initiation codon ATG>AGG, IVS-I-5 (G>C), codon 31 (‒C), −30 (T>C), and −32 (C>A) were not found in our study.

The very variable α-globin alterations in combination with β-globin mutations were found in 313 cases, and 57 genotype combinations of both Hb disorders were identified (Table 3). --^SEA^/αα combined with β^CD41-42^/β^N^ was identified in 83 cases, which was the most common combination, and the other combinations found are as follows: --^SEA^/αα combined with β^−28^/β^N^, --^SEA^/αα combined with β^IVS−II−654^/β^N^, and -α^3.7^/αα combined with β^CD41-42^/β^N^ (Table 3). Out of 313 cases of α-thal/β-thal, 30 cases were with at least three globin mutations, one case was with one α-globin mutation combined with two β-globin mutations, 28 cases were with two α-globin mutations combined with one β-globin mutation, and one extreme patient had four globin mutations with a genotype of --^SEA^/α^WS^α combined with β^CD41-42^/β^−28^ (Table 3).

As for the frequency of a specific type of mutation in all *α* (or β)-mutant chromosomes in this study population, 18 kinds of mutations were identified, including six *α*-gene mutations and 12 β-gene mutation (Tables 4, 5). The --^SEA^ deletion was the most frequent, accounting for 63.63% of all *α*-mutant chromosomes; the following mutations were found: -α^3.7^, -α^4.2^, α^WS^, and α^CS^, with allele frequencies of 19.54%, 7.47%, 3.56%, and 3.30%, respectively (Table 4). Regarding β-globin mutant chromosomes, five mutations accounted for 94.16% of all β-thalassemia defects; these mutations, in the order of frequency, were CD41-42 (-CTTT) (43.76%), IVS-II-654(C>T) (22.26%), −28 (A>G) (15.36%), CD17 (A>T) (8.76%), and CD71-72 (+A) (4.25%). The other seven mutations with frequencies no more than 2% were −29 (A>G), CD27-28 (+C), CD26 (Hb E) (G>A), IVS-I-1 (G>T), CD43 (G>T), CD14-15 (+G), and Cap (Table 5).

**TABLE 4:** Allele frequency of α-thal in individuals of Yangjiang region.

Mutation	HGVS name	Phenotype	Allele (n)	Frequency (%)
--^SEA^	NG_000006.1:g.26264_45564del19301	α^0^	3823	63.63
-α^3.7^	NG_000006.1:g.34164_37967del3804	α^+^	1174	19.54
-α^4.2^	AF221717	α^+^	449	7.47
α^WS^	HBA2:c.369C>G	α^+^	214	3.56
α^QS^	HBA2:c.377T>C	α^+^	150	2.50
α^CS^	HBA2:c.427T>C	α^+^	198	3.30
Total			6008	100

**TABLE 5:** Allele frequency of β-thal in individuals of Yangjiang region.

Mutation	HGVS name	Phenotype	Allele (n)	Frequency (%)
CD41-42	HBB: c.126_129delCTTT	β^0^	1034	43.76
IVS-II-654	HBB: c.316–197C>T	β^+^	526	22.26
−28	HBB: c.-78A>G	β^+^	363	15.36
CD17	HBB: c.52A>T	β^0^	207	8.76
CD71-72	HBB: c.216_217insA	β^0^	95	4.02
−29	HBB: c.-79G>A	β^+^	24	1.02
CD27-28	HBB: c.84_85insC	β^0^	31	1.31
CD26	HBB: c.79G>A	β^+^	40	1.69
IVS-I-1	HBB: c.92 + 1G>T	β^0^	25	1.06
Cap	*HBB*: c.-11_-8delAAAC	β^+^	2	0.08
CD14-15	HBB: c.45_46insG	β^0^	4	0.17
CD43	*HBB*: c.130G>T	β^0^	12	0.50
Total			2363	100

Furthermore, a small number of cases suspected with rare genotypes were referred to undergo gene sequencing or gap-PCR (Liang et al., 2022). Rare thalassemia genotypes were identified in 19 cases which were not included in our detection kit; they included four types of rare α-globin genotypes of αα/--^THAI^ (two cases), HKαα/--^SEA^ (four cases), α^WS^α^CD74 GAC>CAC^/-α^4.2^ (two cases), and α^CD31 AGG>AAG^α/--^SEA^ (one case). Six rare β-globin genotypes were identified: CD39 CAG>TAG (one case), IVS-Ⅱ-2 (−T) (two cases), −90(C>T) (three cases), Chinese ^G^γ^+^(Aγδβ)0 (two cases), CD104 (-G) (one case), and CD19 (A>G) (one case) (Table 6).

**TABLE 6:** Summary of rare thalassemia genotypes.

Genotype	Case	Phenotype	HGVS name	Reporter
α-thalassemia				
--^THAI^/αα	2	α^0^/α	NG_000006.1:g.10664_44164del33501	Fischel-Ghodsian et al. (1988)
HKαα/--^SEA^	4	α/α^0^		Wang et al. (2005)
α^WS^α^CD74(GAC>CAC)^/-α^4.2^	2	α+/α+	HBA1:c.223G>C	
αα^CD31(AGG>AAG)^/--^SEA^	1	α+/α^0^	HBA2:c.95G>A	Chen et al. (2000)
β-thalassemia				
β^CD19(AAC>AGC)^/β	1	β^+^/β^Ν^	HBB: c.59A>G	Yang et al. (1989)
β^−90(C>T)^/β	3	β^+^/β^Ν^	HBB: c.-140C>T	Faustino et al. (1992)
β^IVS−Ⅱ−2 (−T)^/β	2	β^0^/β^Ν^	HBB: c.315+2del	Ma et al. (2000)
Chinese (^A^γδβ)^0^/β	2	Gγ(Aγδβ)0/β^Ν^	NG_000007.3:g.48795_127698 del78904	Jones et al. (1981)
β^CD39(CAG>TAG)^/β	1	β^0^/β^Ν^	HBB: c.118C>T	Orkin and Goff (1981)
β^CD104(−G)^/β	1	β^0^/β^Ν^	HBB: c.312delG	Lahr et al. (2007)
Total	19			

## 4 Discussion

Thalassemia is an autosomal recessive hematologic disease, in which one of the genes that encode for the hemoglobin components, the α- and β-globin chains, is reduced or deleted; its clinical phenotype ranges from asymptomatic forms to fatal hemolytic anemia. The globin genes can be affected by a variety of alterations, and two main types were α- and β-thalassemia (α-thal and β-thal). Thalassemia is prevalent in Middle East, Mediterranean countries, Central Asia, India, and southern China, as well as countries along the north coast of Africa and in South America; about 5% of the world’s population is a carrier of thalassemia (Weatherall, 1981; Weatherall, 1997; Lai et al., 2017). Previous studies indicated that there was a high frequency of thalassemia cases in southern China, especially in Guangxi, Guangdong, Jiangxi, and Hainan provinces (Xu et al., 2004; Xiong et al., 2010; Lin et al., 2014; Yao et al., 2014; Yin et al., 2014; Lai et al., 2017; Liang et al., 2022). Patients of each ethnic population carry their own specific types of mutations, including a few very common ones and a variable number of rare ones.

A county in Yangjiang has a high prevalence of thalassemia, which has become a major social and medical concern (Yin et al., 2014). In this study, we performed genetic diagnosis to determine the spectrum of α-thal and β-thal among patients in Yangjiang and set up the foundation for genetic counseling and performing comprehensive care programs. Our study showed that the most common mutation causes of α-thal in Yangjiang were --^SEA^, -α^3.7^, -α^4.2^, and α^CS^. A similar distribution was found in Guangdong, Jiangxi, whole Guangxi Province, and Meizhou and Chaoshan of eastern Guangdong Province (Xu et al., 2004; Xiong et al., 2010; Lin et al., 2013; Lin et al., 2014; Zheng et al., 2016). This pattern was different from that of Hainan Li with fewer --^SEA^ and that of Yunnan with fewer -α^4.2^ (Zhang et al., 2012; Yao et al., 2014).

β-thalassemia is very heterogeneous, both in the molecular defects and the clinical manifestations. More than 200 different mutations in the *β*-*globin* gene have been characterized worldwide (Lai et al., 2017). In this study, of the 17 common mutations in Chinese people, we detected 12 mutations in the Yangjiang population. The three common mutations 41/42 (-TCTT), IVS-II-654 (C>T), and −28 (A>G) accounted for 81.11% of total β-globin gene mutations. We compared the genotype distribution of β-thal mutations in this study with data reported in the population of other areas in China. IVS-II-654(C>T) and 41/42 (-TCTT) were also the most common mutations in Guangdong, mainland China, Jiangxi, and Han nationality in Hainan (Lin et al., 2014; Yao et al., 2014; Yin et al., 2014; Lai et al., 2017). Different from other areas, 41/42 (-TCTT) and CD17 (A>T) were the most common β-thal mutations in Guangxi Province, whereas Hb E and codon 17 (A>T) were the dominant genotypes in Yunnan (Zhang et al., 2012) and codon 17 (A>T) and 41/42 (-TCTT) were the dominant genotypes in Guizhou (Huang et al., 2013). 41/42 (-TCTT) was the only β-thal mutation in Li people of Hainan Province (Yao et al., 2014). β-thal genotypes are relatively population-specific, and each ethnic group has its own set of common mutants (Lai et al., 2017).

Hemoglobin (Hb) Malay was first described in the Malaysian population in 1989 (Yang et al., 1989). Hb Malay results from an AAC→AGC (HBB: c.59A>G, p.Asn19Ser) mutation in codon 19 of the *β*-*globin* gene. This mutation creates an alternate splicing site between codons 17 and 18, reducing the efficiency of the β-chain globin to about 60% of the normal level (Yang et al., 1989). Hb Malay carriers do not present any clinical symptoms, only present increased HbA2 and decreased MCV, and are often diagnosed during thalassemia screening. However, compound heterozygosity for Hb Malay and β^0^-thal mutation shows β-thal intermedia clinically (Yang et al., 1989). CD104 (-G) (HBB: c.312delG), a heterozygous frameshift mutation in exon 2 of the *β*-*globin* gene, results in a dominantly inherited β^0^-phenotype with mild anemia (Lin et al., 2017). To date, there has been no report of CD104 (-G) and Hb Malay in mainland China in the literature.

Hb H disease was diagnosed in 341 patients, and β-thal major or intermedia were found in 16 patients in this study. Our results revealed that the public health burden imposed by the high prevalence of thalassemia was still severe in Yangjiang. As we all know, the only curative therapy for β-thalassemia major is allogeneic hematopoietic stem cell transplantation; however, the difficulty to find matched donors and economic burden have limited this therapeutic option (Zhou et al., 2008). Due to the large number of thalassemia patients and limited medical resources, it is difficult to give regular blood transfusions to the majority of patients. Therefore, the most effective strategy for this area is to develop an effective prevention program, which includes carrier screening, prenatal diagnosis, and genetic counseling for the couples at risk of giving birth to new cases of thalassemia.

## 5 Conclusion

In conclusion, --^SEA^ was the most common *α*-allele in Yangjiang, and the following alleles were found: -α^3.7^, -α^4.2^, α^CS^, α^WS^, and α^QS^. CD41/42 (−TCTT), IVS-II-654(C>T), and −28 (A>G) were the main alleles of all β-thal alleles. This study provided detailed genotypes of thalassemia in Yangjiang of western Guangdong Province and reflected the complexity of genotypes in this high-prevalence region, and this would be valuable for diagnosis and counseling for thalassemia in this area.

## Data Availability

The raw data supporting the conclusion of this article will be made available by the authors, without undue reservation.
